# Regulation of the expression level of transcription factor XylS reveals new functional insight into its induction mechanism at the *Pm* promoter

**DOI:** 10.1186/1471-2180-13-262

**Published:** 2013-11-19

**Authors:** Friederike Zwick, Rahmi Lale, Svein Valla

**Affiliations:** 1 Department of Biotechnology, Norwegian University of Science and Technology, Sem Sælands Vei 6/8, Trondheim N-7491, Norway

**Keywords:** XylS, *Pm*, Recombinant gene expression, Induction ratio

## Abstract

**Background:**

XylS is the positive regulator of the inducible *Pm* promoter*,* originating from *Pseudomonas putida,* where the system controls a biochemical pathway involved in degradation of aromatic hydrocarbons, which also act as inducers. The XylS/*Pm* positive regulator/promoter system is used for recombinant gene expression and the output from *Pm* is known to be sensitive to the intracellular XylS concentration.

**Results:**

By constructing a synthetic operon consisting of *xylS* and *luc*, the gene encoding luciferase, relative XylS expression levels could be monitored indirectly at physiological concentrations. Expression of XylS from inducible promoters allowed control over a more than 800-fold range, however, the corresponding output from *Pm* covered only an about five-fold range. The maximum output from *Pm* could not be increased by introducing more copies of the promoter in the cells. Interestingly, a previously reported XylS variant (StEP-13), known to strongly stimulate expression from *Pm*, caused the same maximum activity from *Pm* as wild-type XylS at high XylS expression levels. Under uninduced conditions expression from *Pm* also increased as a function of XylS expression levels, and at very high concentrations the maximum activity from *Pm* was the same as in the presence of inducer.

**Conclusion:**

According to our proposed model, which is in agreement with current knowledge, the regulator, XylS, can exist in three states: monomers, dimers, and aggregates. Only the dimers are active and able to induce expression from *Pm*. Their maximum intracellular concentration and the corresponding output from *Pm* are limited by the concentration-dependent conversion into inactive aggregates. Maximization of the induction ratio at *Pm* can be obtained by expression of XylS at the level where aggregation occurs, which might be exploited for recombinant gene expression. The results described here also indicate that there might exist variants of XylS which can exist at higher active dimer concentrations and thus lead to increased expression levels from *Pm*.

## Background

Regulated promoters are commonly used in recombinant protein production processes and are particularly important for production of host-toxic proteins or proteins that cause a serious metabolic burden to the host cells [1,2]. The transcription regulator XylS stimulates expression from the *Pm* promoter in the presence of benzoic acid or derivatives thereof [3]. XylS originates from the *Pseudomonas putida* TOL-plasmid and is expressed from two different promoters, *Ps1* and *Ps2: Ps1* is regulated, while *Ps2* is constitutive [4]. The production level of XylS from *Ps2* is low, leading to an estimated amount of about 200 molecules per cell [5].

XylS belongs to the AraC/XylS family of transcription factors and it has been shown to be transcriptionally active as a dimer. Dimerization occurs both in the absence and presence of inducer, but to a greater extent in its presence [5,6]. In spite of sequence similarities and common functional domains, the different members of the AraC/XylS family act via a range of different mechanisms. AraC, for example, forms dimers like XylS, both in the presence and absence of inducer [7]. In the presence of inducer it acts as an activator of gene expression (like XylS), but in the absence of inducer, it represses gene expression via DNA bending. The first two proteins of the AraC/XylS family, for which 3D crystal structures have been determined, were RobA and MarA, and both exist as monomers only [8].

XylS consists of two domains and structural models exist for both, constructed based on sequence alignments [9,10]. The model of the N-terminal domain proposes a β-barrel, which is involved in inducer binding and two α-helices that probably are involved in dimerization [10-12]. In the C-terminal domain seven α-helices that form two helix-turn-helix motifs are proposed [9]. These motifs are responsible for binding to two direct repeats with the sequence TGCAN_6_GGNTA upstream of the -35 box of *Pm*[13,14]. The second binding site overlaps by two bases with the -35 box and this overlap is essential for transcription initiation from *Pm*[15]. Both domains are thought to interact with the host RNA polymerase (RNAP) [16-19].

The N-terminal domain has been shown to suppress the action of the C-terminal domain in the absence of inducer [5,20]. Binding of wild type XylS to DNA can only be observed when the protein is dimerized [5]. A XylS-variant which is unable to form dimers has been shown to bind to DNA only in the presence of inducer, which leads to the same conformational changes as dimerization, however, this particular variant is unable to activate expression from *Pm*[5]. Recently it has been shown that XylS dimers bind to DNA sequentially. The first monomer to bind is the one proximal to the RNAP binding site. This leads to [10DNA bending, which in turn enables the second monomer to bind, and indicates that XylS is dimerized prior to DNA binding [16]. At typical cell-internal XylS-levels only 30-40% of the *Pm* promoter sequences are occupied *in vitro* and it has been proposed that complete occupancy cannot be achieved by XylS amounts which do not exceed its intracellular solubility [21].

Vectors which combine the XylS/*Pm* expression system with the broad-host-range mini-RK2 replicon [22,23], in which XylS is expressed from its natural *Ps2* promoter, have been shown to be capable of producing recombinant proteins at industrial levels in *Escherichia coli*[24,25]. Expression levels of these vectors could be heavily increased by mutating different DNA control elements of the expression cassette [10,26,27], and recently it has been demonstrated that they could be yet further improved when mutated DNA elements were combined [28]. When induced expression levels are increased it leads, in most cases, to undesired high expression levels also in the absence of inducer. For the XylS/*Pm* expression system the background expression could be strongly reduced when the 5′-UTR flanking the Shine-Dalgarno site was mutagenized and this has been demonstrated to be useful for metabolic engineering purposes [29]. With this approach an induction ratio of 260-fold could be reached, however, as a consequence induced expression levels were also reduced for these constructs. A possible alternative method of reducing uninduced expression could be to regulate the XylS expression level. Previous experiments have shown that strong XylS overexpression, as for example from the bacteriophage T7 promoter or from *Ps1*, results in a complete loss of inducibility [21,30]. Fusion of *xylS* to the *Psal* promoter, which can be activated by similar inducers as *Pm*, allowed simultaneous induction of XylS expression and XylS activation. Induction ratios that could be reached by this approach were about 180- to 240-fold [31].

Here we report a more detailed study on the relationship between XylS expression levels and expression levels achieved from the *Pm* promoter, both under induced and uninduced conditions. Based on the outcomes of this study we propose a model that aims to explain the behaviour of XylS as a function of its concentration and its formation of monomers, dimers and higher order oligomers.

## Results and discussion

### Construction of a synthetic operon that can be used to indirectly measure relative XylS expression levels

**Figure 1:**
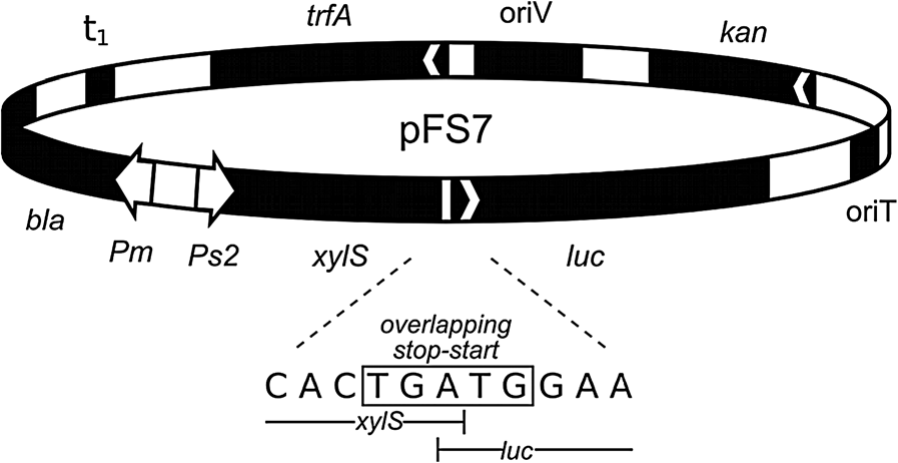
**Map of plasmid pFS7.***Ps2*: constitutive promoter; *xylS*: gene encoding *Pm* activator; *luc*: gene encoding luciferase; *Pm*: positively regulated promoter; *bla*: ampicillin resistance gene encoding β-lactamase; *t*_*1*_: *rrnBT*_*1*_*T*_*2*_ bidirectional transcriptional terminator; *trfA*: gene encoding the replication protein; *oriV*: origin of vegetative replication; *kan*: kanamycin resistance gene; *oriT*: origin of conjugal transfer. The DNA sequence of the overlapping stop-start codon is depicted.

**Figure 2:**
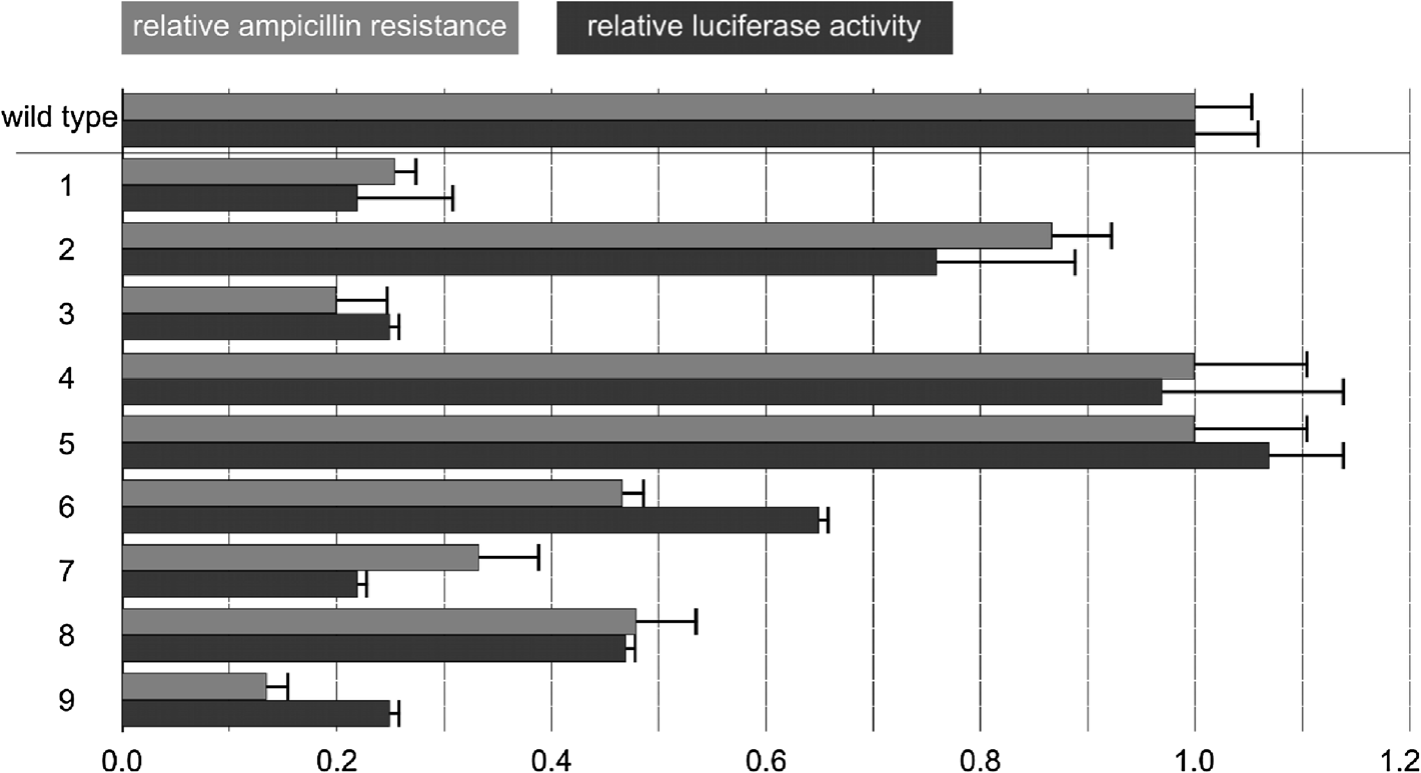
**Expression levels from pFS7 for different variants of *xylS *with silent mutations.** Relative expression levels from *Pm* (measured as maximum ampicillin tolerance at 1 mM *m*-toluate) are given in grey (error bars = lowest ampicillin concentrations in test on which no growth was observed) and relative luciferase activity as a measure for XylS amounts in black (values from at least two biological replicas). All values (relative ampicillin tolerance and luciferase expression) refer to those of wild type XylS (tolerating 350 μg mL^-1^), which are both arbitrarily set to 1. Mutations in the variants (1 to 9), the number stands for the base position that has been changed, relative to the translational start site, the character tells the base in the variant. 1: 6- > C; 2: 13- > C; 3: 15- > G; 4: 16- > C; 5: 27- > G; 6: 30- > C; 7: 36- > T; 8: 42- > T; 9: all of the eight mutations.

With the goal to enable detection of XylS at low concentrations we developed a synthetic operon in which luciferase functions as an indirect indicator of expression of XylS from its native *Ps2* promoter. In this design the *luc* gene is transcriptionally fused to *xylS* via overlapping stop and start codons and should be translated only when *xylS* is translated first. The new plasmid was designated as pFS7 (Figure 1). To test the functionality of this construct we used a series of *xylS* variant sequences which had been synthesized. These variants contain synonymous codon changes relative to the wild type sequence and had been found to activate *Pm* to varying extents (in the presence of induction). We hypothesized that the effects of the codon changes were caused by variations in *xylS* mRNA translation, since transcript amounts were found to be similar to the levels of the wild type gene (qRT-PCR, data not shown). Nine such variant sequences were tested in pFS7, and luciferase activities were measured (Figure 2). The values varied in the range from about 20 to 100% of that of the construct containing the wild type *xylS*.

The design of plasmid pFS7 also allowed us to study the effects of the changed XylS expression on activation of *Pm*. For this purpose the *bla* gene, encoding β-lactamase, was used as a reporter (see Figure 1). We have previously used this gene to monitor expression from *Pm,* since the tolerance of the host to ampicillin correlates well with the produced amounts of β-lactamase in a directly proportional way [32], up to ampicillin concentrations of 16 mg mL^-1^, thus making it easier to identify clones with desired phenotype without laborious library screening [10,26,27]. The data for luciferase activities and host ampicillin tolerances on agar medium correlated well (Figure 2). As an additional control we compared the ampicillin tolerances of all the nine constructs (and wild type) to those in plasmid pTA13 (similar to pFS7, but without *luc*), and found that the relative maximum ampicillin tolerances between the corresponding hosts were essentially the same (data not shown). These results indicate that luciferase activities reflect the levels of XylS expression in the cells, and that the activity of *Pm* also correlates with XylS expression, at least at these physiological and low concentrations.

### In trans activation of expression from Pm by XylS increases the induction ratio

The XylS concentrations that could be generated via synonymous codon variants spanned only a five-fold range, and none of the expression levels were significantly higher than that of the wild type *xylS* gene (Figure 2). To expand the concentration range and increase the maximum level of expression from *Pm,* we expressed XylS *in trans* from a separate plasmid compatible with pFS7. This plasmid was based on the pBBR1 replicon (about five-fold higher copy number than the mini-RK2 replicons) and the *xylS* gene under its native *Ps2* promoter (as in pFS7) was inserted, generating pFZ2A. The *xylS* and *luc* genes were deleted from plasmid pFS7 leading to pFS15. Maximum ampicillin tolerances of cells containing both pFZ2A (expressing *xylS-luc*) and pFS15 (harboring *Pm*) were approximately 5 μg mL^-1^ (uninduced) and 2500 μg mL^-1^ (induced with 1 mM *m*-toluate), which gives rise to an induction ratio as high as about 500-fold. The increase in ampicillin tolerance in the presence of *m*-toluate, compared to the setting where XylS is expressed *in cis* (pFS7, 350 μg mL^-1^), was not unexpected and might be explained by the higher copy number of plasmid pFZ2A relative to pFS7, leading to more XylS expression. In contrast, the uninduced background level (expression from the promoter in the absence of induction) remained significantly lower in the *trans* situation than in the *cis* situation, in fact it was similar to the cellular background tolerance in the absence of any plasmid. This phenomenon might be explained by the fact that XylS will dimerize only occasionally in the absence of inducer. Probably the concentration of XylS and consequentially also dimers of the protein is highest near the site of synthesis. The larger spatial distance from *Pm* in the *trans* situation will then lead to a lack of dimers at the promoter site. In the *cis* situation the chance of XylS dimers to bind to *Pm* will be higher, as the protein is produced in close proximity to the promoter. The lower background level in the *trans* situation may be of practical interest, for example in cases where expression from *Pm* is maximized by mutations in the expression cassette [28], and especially for expression of toxic proteins. In the context of the experiments reported here, the *in trans* system seems clearly well suited for more in-depth studies of the relation between XylS expression and activation of *Pm* in the absence and presence of induction.

### Use of the regulated Pb promoter to control the xylS expression level

**Figure 3:**
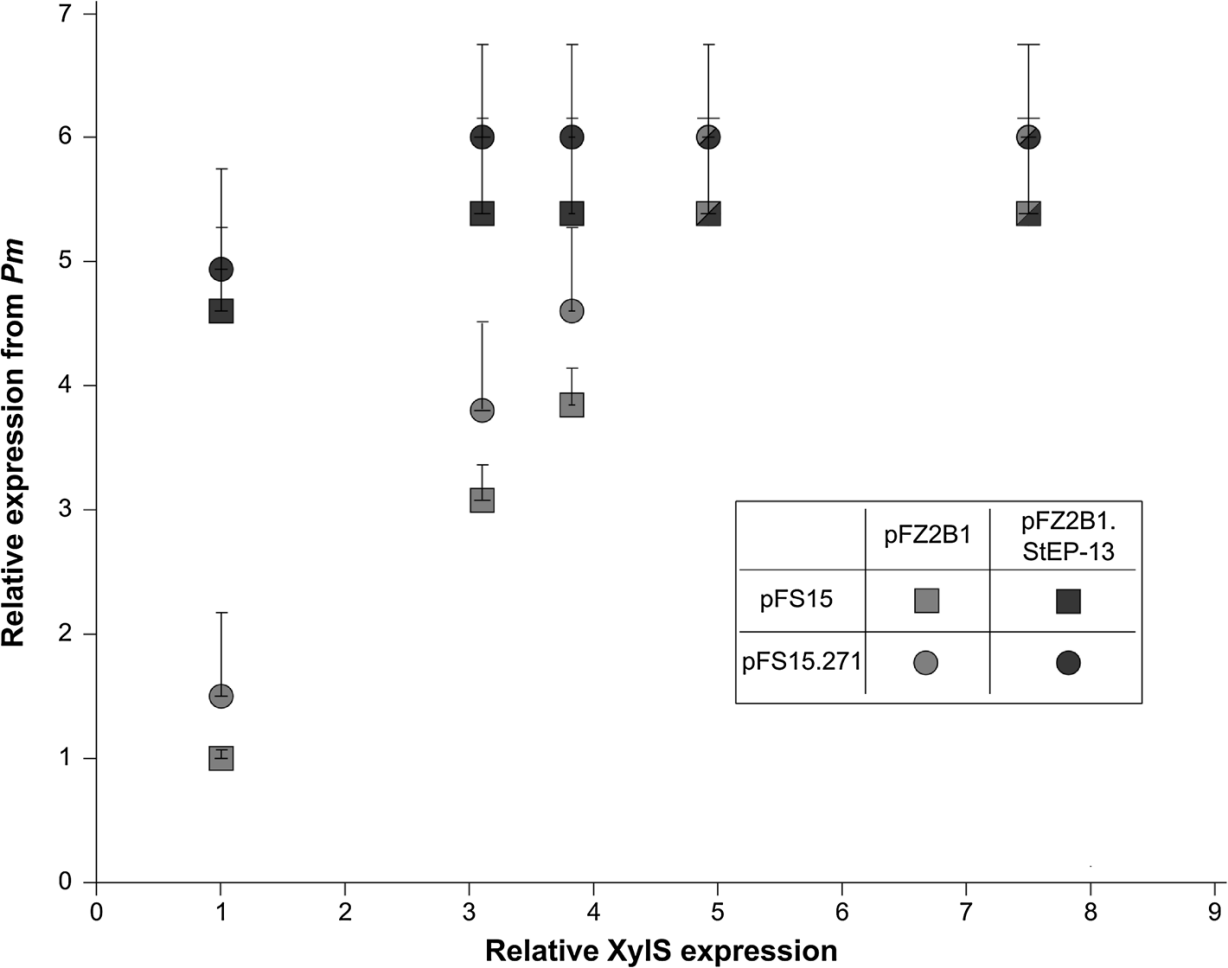
**Effects of variations in wild type or variant XylS expression on *Pm *activity.** Upper host ampicillin tolerance levels as a function of the expression level of wild type XylS (pFZ2B1) or variant StEP-13 (pFZ2B1.StEP-13), using two different copy number variants (pFS15 and pFS15.271) of the target plasmid. *Pm* activity was measured as upper relative ampicillin tolerance on agar medium. The tolerance for cells containing pFZ2B1 + pFS15, no cyclohexanone, was arbitrarily set to 1 and corresponds to about 650 μg mL^-1^ ampicillin resistance. The relative XylS expression was measured as luciferase activity and was also set to 1 for the same data point. The data points indicate the highest ampicillin concentration on which growth occurred, while the lowest concentration on which no growth was observed is indicated by error bars. Shapes that are half grey and half black indicate identical data points for both wild type and StEP-13. 1 mM *m*-toluate was added to all samples, cyclohexanone concentrations leading to the measured XylS expression levels (from left to right): 0, 0.25, 0.5, 1 and 2 mM, respectively.

The experiments described above as well as previously published studies [21,31] demonstrate that expression from *Pm* can be increased by producing more XylS, and to determine what the maximum level is we decided to use the inducible *Pb* promoter from *Acinetobacter* sp*.* to express XylS*. Pb*, like *Pm,* can be used to regulate expression of genes in a continuously graded manner [33]. It is positively regulated by the ChnR protein, which also belongs to the AraC/XylS transcription factor family, in the presence of its inducer cyclohexanone. The *xylS-luc* operon expressed from *Pb* and the gene of the activator protein, *chnR*, were cloned into pBBR1MCS-5 [34], generating pFZ2B1, and pFS15 was used as target plasmid for XylS harboring the *Pm* promoter, as described above. Cells containing both of these plasmids were plated on agar medium, supplemented with varying amounts of ampicillin, cyclohexanone and *m*-toluate. As expected, cells with only one of the two plasmids (either pFZ2B1 or pFS15) reacted only marginally to the addition of the inducers. However, in the presence of both plasmids the ampicillin tolerance of the host cells varied as a function of both the cyclohexanone and *m-*toluate concentrations. At a fixed 1 mM m-toluate concentration the host ampicillin tolerance correlated well with both the concentration of cyclohexanone and the luciferase activity, which reflects XylS expression (Figure 3, grey squares). However, at the two highest concentrations of cyclohexanone tested (1 and 2 mM) the upper ampicillin tolerances were similar (3500 μg mL^-1^) and about 5.4 times higher than in the absence of the *Pb* inducer.

**Figure 4:**
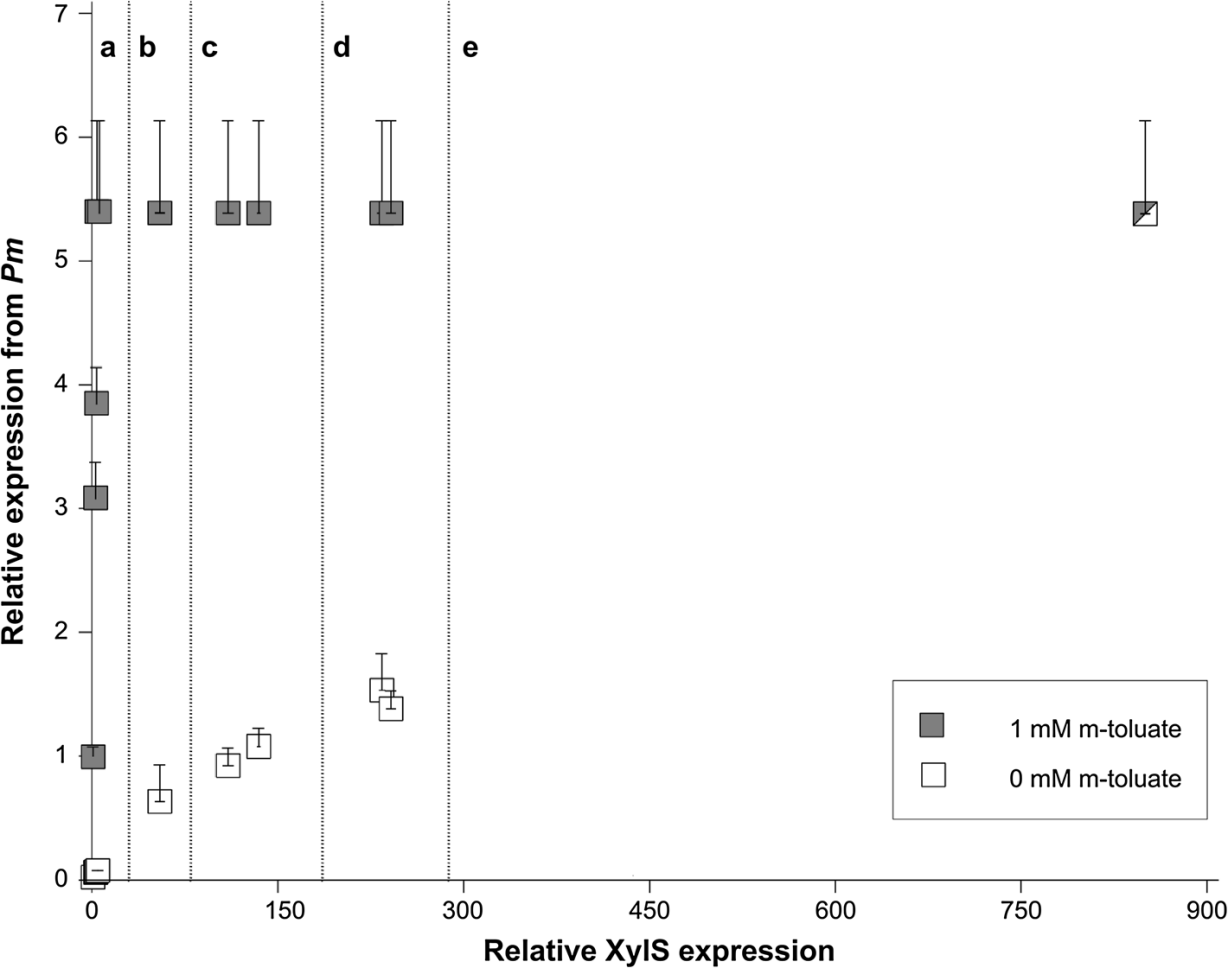
**Effects of XylS expression variations on induced and uninduced *Pm *activity.** Upper host ampicillin tolerance levels as a function of the expression level of XylS in the absence (white squares) and presence (grey squares) of *Pm* induction (0/1 mM *m*-toluate). The shape that is half grey and half white represents an identical data point for both induced and uninduced. Relative expression from *Pm* and relative XylS expression were determined in the same way as described in Figure 3. The data points were collected from cells containing the *Pm*-bearing plasmid pFS15 in all cases and **a**: pFZ2B1, inducer concentrations as in Figure 3 (the grey data points are the same as the corresponding points in Figure 3); **b**: pET16.xylS, 0 mM IPTG; **c**: pFZ2B2, 0.25 and 0.5 mM cyclohexanone (from left to right); **d**: pFZ2B3, 0.25 and 0.5 mM cyclohexanone (from left to right); **e**: pET16.xylS, 0.5 mM IPTG.

The results presented above might indicate that expression from *Pm* could not be stimulated more by further increasing the XylS expression. We have on the other hand observed that 2 mM cyclohexanone is not so far from concentrations that have observable negative effects on cell growth [34], and we therefore wanted to create conditions at which XylS expression could be increased further without using near-toxic concentrations of cyclohexanone. In a parallel ongoing project we had observed that the expression level from the *Pb* promoter is, like *Pm*, very sensitive to the amounts of its regulator, ChnR. This was taken advantage of by substituting the *chnR* native promoter with constitutive promoters from the Registry of Standard Biological Parts, which were identified by a library screening [35]. Two promising variants were used to drive *chnR* expression in derivatives of pFZ2B1, namely pFZ2B2 and pFZ2B3, such that XylS expression could be controlled by cyclohexanone, as above, but hopefully at higher levels. As expected this resulted in increased XylS expression (measured as luciferase activity), up to 50-fold (pFZ2B3) above the maximum for pFZ2B1. In spite of this, the expression from *Pm* (in pFS15) was not higher than when pFZ2B1 was used for expression of XylS (Figure 4a,c and d, grey squares).

For studies of expression from *Pm* in the absence of *m*-toluate (see further down) we also expressed *xylS* from the very strong bacteriophage T7 promoter (in plasmid pET16.xylS), heavily used for recombinant protein production. Activation of the T7 promoter requires the presence of T7 RNAP, and its production is induced by isopropyl β-D-1-thiogalactopyranoside (IPTG). In the presence of this inducer XylS expression (measured as luciferase activity) was increased about five-fold compared to the maximum achieved by pFZ2B3, but the corresponding host tolerance to ampicillin did not increase any further (Figure 4e). Since we know that the ampicillin tolerance of the host can be increased to much higher levels by other means [28] we concluded that by using pFS15 as target plasmid for XylS, expression of the reporter (*bla*) downstream of *Pm* cannot be increased beyond the identified maximum (3500 μg mL^-1^) by enhancing production of XylS.

### The XylS variant StEP-13 stimulates expression from Pm to the same maximum level as wild type XylS

In a previous study in our laboratory variants of *xylS* were isolated that resulted in strongly stimulated expression from *Pm*[10]. One such variant (StEP-13), which contains five amino acid substitutions (F3Y, I50T, F97L, E195G, M196T [10]) and originated from a combination of error-prone PCR and DNA shuffling procedures, was subjected to a comparative analysis with wild type *xylS*. This was done by first substituting the wild type *xylS* in pFS7 with the variant gene. Both *xylS* transcript amounts and luciferase activity were found to be the same for the resulting plasmid as for pFS7 (data not shown), indicating that the XylS expression level was not affected by the mutations in StEP-13. Thus it was concluded that StEP-13 increases expression from *Pm* via modified functionality of the protein.

To study expression from *Pm* as a function of expression of StEP-13, this particular variant was placed under control of the *Pb* promoter in plasmids analogous to pFZ2B1 and pFZ2B3 (pFZ2BX.StEP-13) and transformed into cells also containing pFS15. At low regulator expression levels cells with StEP-13, as expected, conferred an in general higher ampicillin tolerance than cells with wild type XylS (see Figure 3, grey and black squares). More interestingly, the same maximum level of resistance as for wild type XylS was observed, albeit it was reached at lower regulator concentrations. No changes in maximum resistance were found for host cells containing pFZ2B3.StEP-13 either (data not shown). This implies that the variant StEP-13 increases expression from *Pm* only at sub-saturating concentrations. All mutations in StEP-13 are situated in its N-terminal domain, while the C-terminal domain is involved in DNA binding. Thus it is reasonable to assume that StEP-13 acts either via better inducer binding, increased dimerization (which also can be a consequence of better inducer binding), stronger interaction with the host RNAP or a combination of these. Improved inducer binding could be excluded as single explanation for the phenotype of StEP-13, as the variant increases expression from *Pm* quite significantly also in the absence of *m*-toluate (data not shown).

### The observed maximum expression level from Pm is not caused by saturation of available XylS target DNA binding sites

One way of explaining the observed maximum expression level is to assume that at some threshold value the XylS amounts in the cells are sufficient to saturate all the corresponding binding sites upstream of *Pm*. The behavior of StEP-13 could then be explained by a stronger affinity of the variant for binding to *Pm* (for example via improved dimerization), which would lead to a saturation of all binding sites at lower XylS expression levels.

This hypothesis would lead to the assumption that it might be possible to increase expression from *Pm* further simply by raising the copy number of the XylS target plasmid, pFSF15, which can be done by introducing specific point mutations in the gene encoding the replication initiation protein, TrfA. The positive correlation between plasmid copy number and level of recombinant protein expression is well established, and we have also used it specifically for *Pm* in mini-RK2 plasmids [23-25,36]. However, in previous applications the level of XylS expression was not taken into consideration and in all reported experiments the number of *xylS* copies was increased equally to the number of *Pm*. The *trfA* variant cop271 leads to 3-4-fold increased plasmid copy number compared to its wild type equivalent (4–8 copies per chromosome) [37]. This variant was integrated into pFS15 (generating pFS15.271) and transformed into cells, which already harbored pFZ2B1 or pFZ2B1.StEP-13. Host ampicillin tolerance was then monitored as a function of XylS expression (luciferase activity), and the previously observed maximum ampicillin tolerance level was found to increase only marginally, both for wild type XylS and StEP-13, and much less than in proportion to the expected increase in XylS binding sites.

The maximum ampicillin tolerance level also leveled out at similar XylS expression levels as with the wild type copy number (Figure 3, circles). Based on this we concluded that at maximum expression from pFS15 the limiting factor is not the number of target DNA molecules for XylS binding. This is also in agreement with previously published studies, in which the authors concluded that the interactions between XylS and *Pm* are too weak to lead to complete saturation [21]. Since the number of target DNA molecules did not appear to limit the maximum expression level from *Pm* we reasoned that more likely some property of XylS was causing the apparent saturation of the system at a certain concentration of this regulator.

### In the presence of very high XylS concentrations expression from Pm can reach the upper maximum level in the absence of inducer

It is known that *Pm* looses its inducibility at high levels of XylS expression [21,30]. As we now had a way of varying and semi-quantitatively measuring XylS concentrations we could also evaluate the response in the absence of *Pm* inducer (Figure 4, white squares). In the absence of both *m*-toluate and cyclohexanone cells with pFZ2B1 and pFS15 did not tolerate significantly more ampicillin than cells without any plasmid. As expected, the activation of the *Pm* promoter was less sensitive to the presence of cyclohexanone than to the presence of *m*-toluate. This implies that the induction ratio of the system becomes higher as a function of XylS expression levels, up to the point where the maximum expression is observed. A maximum induction ratio of about 700 is reached at this point (about five times more XylS expression than in the absence of cyclohexanone). Interestingly, above this level expression from *Pm* in the absence of *m*-toluate continued to increase as a function of XylS expression levels, and at the highest production achievable with the available production tools (induced T7 promoter, relative XylS expression about 850), the maximum ampicillin tolerance of the host cells was at the same level as in the presence of inducer (Figure 4e).

### XylS is produced from the T7 promoter mainly in an insoluble form

**Figure 5:**
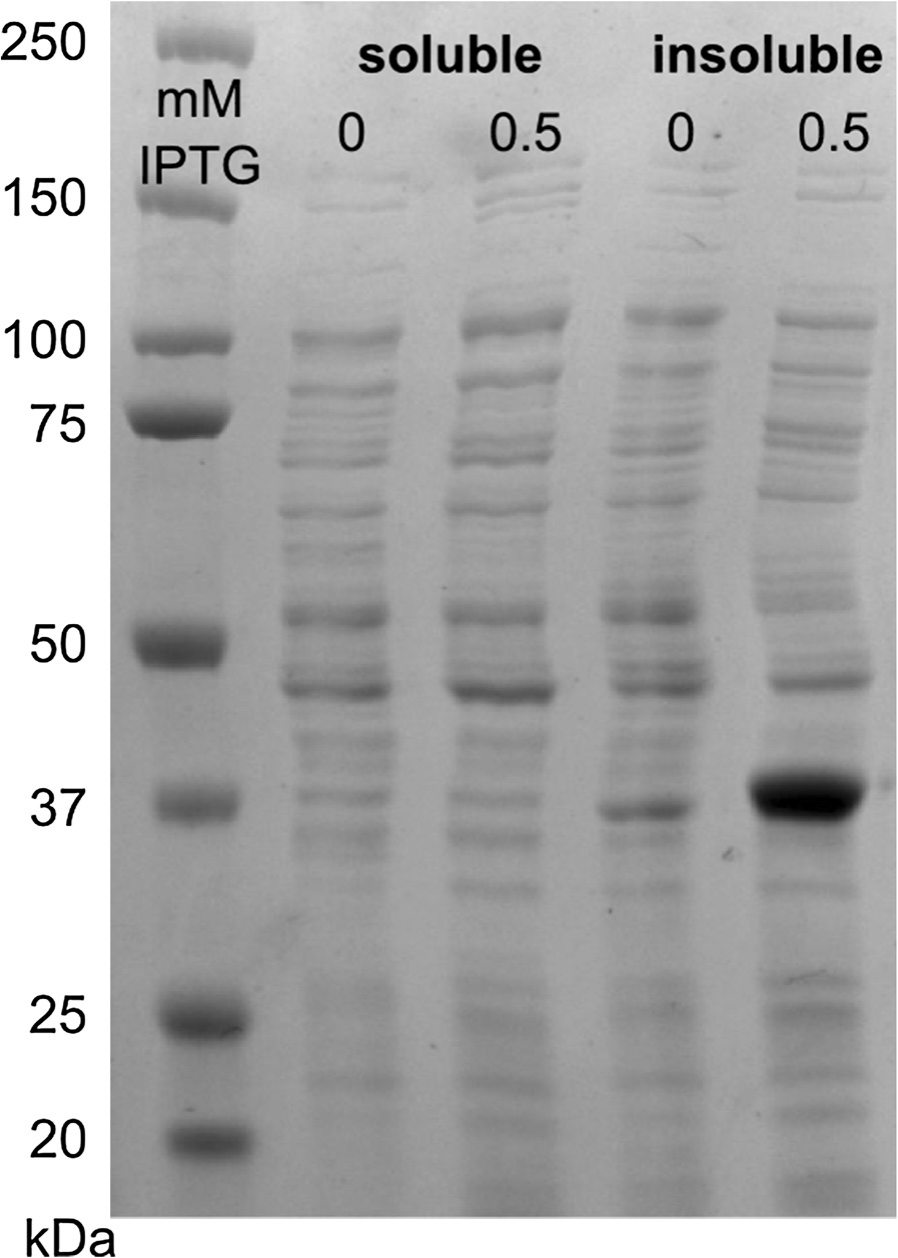
**SDS-PAGE gel for XylS produced from the T7 promoter.** Samples were crude bacterial lysates from cells containing vector pET16b.xylS, grown in the presence or absence of inducer. Samples were split into soluble and insoluble fractions. Sizes of the protein ladder in kDa are given on the left site.

Based on the luciferase activity measurements over 800 times more XylS was expressed from the T7 promoter than from *Ps2*. If previous estimates of about 200 molecules per cell [5] are reasonably close to the true value, simple calculations indicated that an over 800-fold increase would yield a band directly visible on SDS-PAGE. A bacterial cell culture containing plasmid pET16.xylS was split into two such that one was induced by IPTG (0.5 mM), the other was not. Cells were harvested by centrifugation, lysed and split into a soluble and an insoluble fraction by centrifugation and the resulting samples were separated on an SDS-PAGE gel. Inspection of the band patterns (Figure 5) clearly demonstrated a unique and strong band in only the sample from the induced insoluble fraction. The distance of migration also matched to the expected position for XylS (36 kDa). The weaker band representing a similar size protein in the insoluble fraction of the uninduced culture seems to originate from a host-derived protein, as the same band was observed for samples from cells containing plasmid without *xylS* both in the presence and absence of inducer (data not shown). Thus, the vast majority of the XylS protein expressed from pET16.xylS is produced in an aggregated and presumably inactive form.

### Model for activation of Pm by XylS

**Figure 6:**
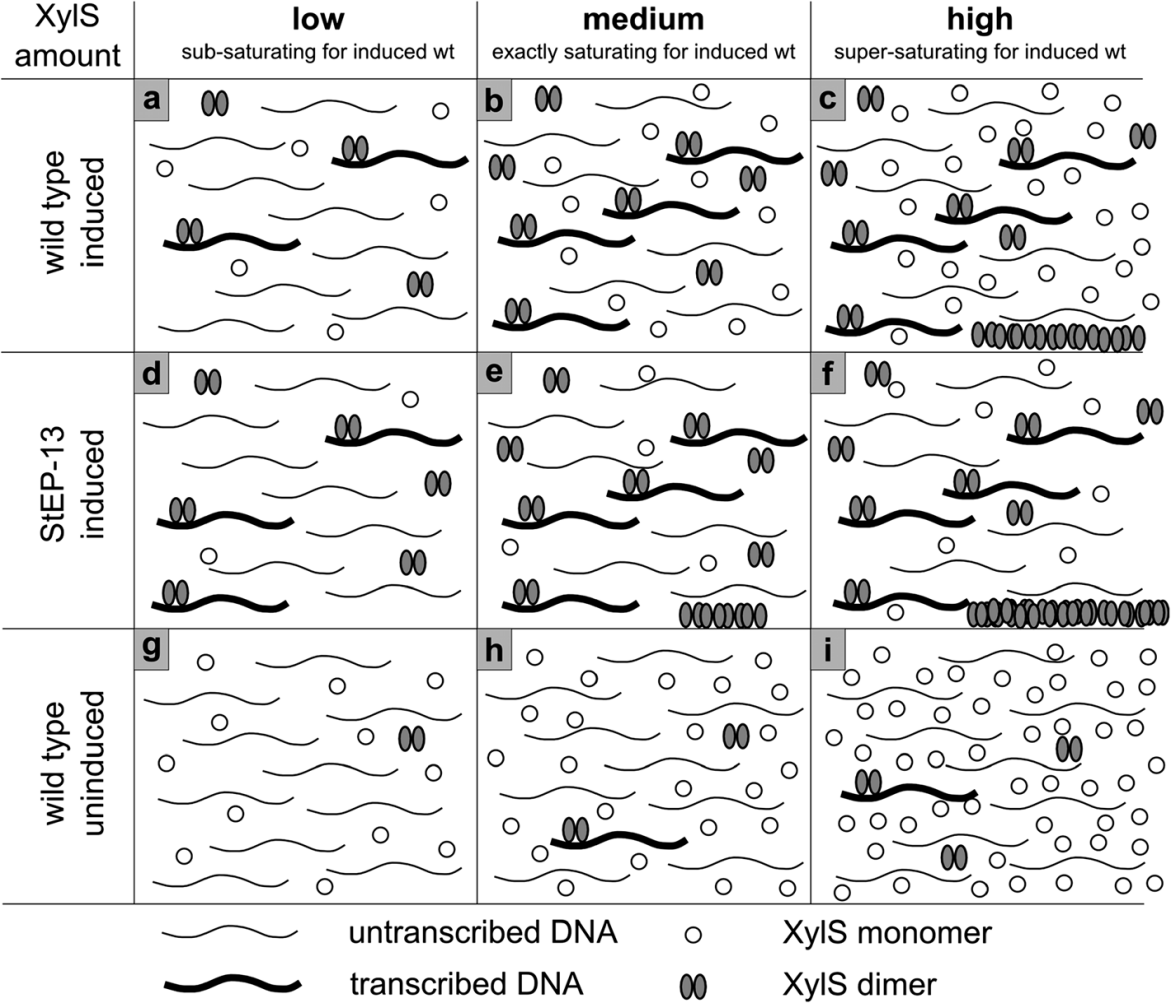
**Visualization of the hypothesis explaining XylS behaviour at various intracellular concentrations.** The numbers of DNA or XylS molecules are not meant to represent the actual numbers in the cells. Only aggregates formed from active dimers of the protein are considered. At low XylS concentrations a certain percentage of the dimerized XylS molecules will activate transcription **(a)**; the amount of activated *Pm* promoters will increase proportionally to XylS amounts up to a certain treshold value **(b)**; when the threshold value is exceeded, XylS dimers will aggregate and become inactive, while the amount of active dimers remains constant **(c)**. For StEP-13 a higher percentage of XylS molecules will dimerize at low XylS concentrations, resulting in more transcribed DNA **(d)**; when the saturating concentration for wild type XylS is reached, there will already be some aggregation of dimers in case of StEP-13 **(e)**, and as for wild type this will increase further as more XylS is expressed **(f)**. In the absence of *m-*toluate, only a very small fraction of the XylS molecules will form dimers and these will activate transcription from *Pm*, aggregation does not start at the XylS expression levels depicted here **(g, h, i)**.

The observations reported here are consistent with and extend previous knowledge related to XylS function, and together they support the following model:

In the absence of *m*-toluate XylS is mainly present in a monomeric state, which probably is not able to activate *Pm*, while in the presence of *m*-toluate an unknown fraction of these monomers are converted to dimers, which activate transcription from *Pm*[5,6]. At low XylS concentrations formation of active dimers probably depends on *m*-toluate concentrations (Figure 6a), and this assumption can explain the well known fact that expression from *Pm* correlates with the concentration of inducer at fixed levels of XylS expression (usually from *Ps2*). In contrast, above a certain threshold value for XylS expression (illustrated in Figure 6b) the activity from *Pm* does not increase any further, and this can be explained by formation of XylS in a third state, as aggregated and not active molecules (Figure 6c). Alternatively, the threshold value might also be caused by saturation of the *Pm* targets available in the cell. However, this explanation does not fit with the observation that introduction of more *Pm* copies does not lead to a corresponding stimulation of expression even if total XylS levels are increased beyond the threshold value (Figure 3). Therefore, the upper maximum level of active dimers in the cells seems to be the result of inherent properties of the XylS molecule itself.

The XylS variant StEP-13 is interesting in that it was previously found to strongly stimulate expression levels from *Pm*, compared to the wild type XylS [10]. In the referred study the regulator was expressed from *Ps2*, now known to produce only sub-saturating concentrations of XylS with respect to activation of *Pm*. It is therefore interesting that the experiments reported here show that when the expression level of StEP-13 was increased the maximum out-put from *Pm* was near the same as for wild type XylS. According to the reasoning above this seems to mean that StEP-13 is not able to form higher concentrations of active dimers than wild type XylS, but it reaches the maximum at lower inducer (*m*-toluate) or regulator concentrations (Figure 6d-e). StEP-13 was generated by complex mutagenesis procedures that may have changed its functional properties in more than one way. This prediction fits with the observation that it responds more efficiently to low inducer concentrations, while it is also more active in the absence of *m*-toluate. Both observations are in agreement with an inherently more efficient ability to form dimers, both in the absence (see below) and presence of *m*-toluate. This could involve higher affinity for the inducer, but no change in the properties related to formation of higher level aggregates from XylS dimers. Thus, more of the dimers would form aggregates at lower total XylS concentrations for StEP-13 than for the wild type protein, while the maximum concentrations of active dimers would be unchanged (Figure 6e-f). Another possibility could be that each dimer interacts more efficiently with RNAP, but one might then predict that the maximum level of expression from *Pm* would also be increased compared to wild type XylS.

The behavior of XylS in the absence of inducer (*m*-toluate) can be explained by the same model (Figure 6g-i). Dimerization of the regulator is strongly stimulated in the presence of inducer, but a certain low fraction of XylS dimerizes also in the absence of inducer. However, much higher total concentrations of the regulator are required before the maximum dimer concentration is reached. As a consequence aggregation will also start at much higher XylS expression levels.

If this model holds true it leads to an interesting prediction that if one could mutagenize *xylS,* such that its protein product could form higher concentrations of active dimers (less aggregate formation), expression from *Pm* could be further stimulated.

A screening for such variants should probably be done under conditions of excessive amounts of XylS present in the cells, to make sure that the desired phenotype is actually detected. StEP-13 was identified while expressed from *Ps2* (and thus at low levels), and other types of variants may then dominate the screening outcome.

Even though XylS is known to be produced at low levels from its natural *Ps2* promoter [5] these small amounts are sufficient for successful applications of *Pm* in recombinant protein production [24,25]. The results reported here indicate that expression can be further stimulated by increasing the intracellular concentration of XylS, and by fine-tuning this level and expressing XylS *in trans* the induction ratio can also be maximized. As shown here this allowed for high expression levels while maintaining an induction ratio of 700-fold, which exceeds the reported induction ratios both reached by 5′-UTR variations [29] and by regulation of XylS expression by a promoter which is activated by the same inducer as *Pm*[31].

In earlier studies a linear correlation between the copy number of plasmids that carry the complete XylS/*Pm* system and expression levels from *Pm* has been observed [23-25]. It is common to assume that this well known effect is caused by increased dosage of the gene to be expressed, but for a given XylS/*Pm*-based system the results presented here indicate that it is the increased amounts of XylS that lead to more expression from *Pm*. Fortunately the performance of the XylS/*Pm* system is not limited exclusively by concentrations of XylS dimers, since expression from *Pm* can be drastically stimulated by using combinations of various types of mutations in the expression cassette [28].

## Conclusions

The earlier reported complete loss of inducibility of *Pm* at high levels of XylS expression [21,30] can be explained by the existence of a maximum concentration of active dimers inside the cell. An increase in XylS amounts beyond the point at which this maximum concentration is reached will lead to the formation of inactive aggregates. For very high cell-internal XylS amounts the concentration of dimers will thus be the same under induced and uninduced conditions. These findings enable expression of the transcription factor at a level for which the induction ratio at *Pm* is maximized, which is of high importance for recombinant gene expression.

## Methods

### Strains and growth conditions

The main bacterial strain used as host in this study was *Escherichia coli* DH5α (Bethesda Research Laboratories), unless otherwise stated. The cells were cultivated at 37°C in Lysogeny Broth (LB) (10 g L^-1^ tryptone, 5 g L^-1^ yeast extract, and 5 g L^-1^ NaCl) or on Lysogeny Agar (LB broth with 20 g L^-1^ agar). Antibiotics concentrations used in this study were: kanamycin 50 μg mL^-1^, gentamicin 20 μg mL^-1^, and tetracycline 15 μg mL^-1^ (final concentration).

For luciferase enzyme assay measurements 10 mL of LB were inoculated from an overnight culture and grown at 37°C to an OD_600_ of 0.1 and then induced with 1 mM *m*-toluate. After induction cells were further incubated at 30°C for 4 hours, before samples were collected.

When the T7 promoter was used, *Escherichia coli* ER2566 (New England Biolabs) was used as a host. Growth conditions were similar to those of DH5α, but for induction IPTG was added to a final concentration of 0.5 mM.

For induction of the ChnR/*Pb* system, cyclohexanone was added at the concentrations indicated.

### Standard DNA manipulations

All enzymes for DNA manipulations were purchased from New England Biolabs and applied as described by the manufacturers. Primers and oligonucleotides were purchased either from Eurofins MWG Operon or Sigma Genosys. Transformations in cloning experiments were performed with a modified RbCl protocol (Promega). For plasmid DNA purifications WizardPlus SV minipreps DNA purification kit (Promega) was used.

PCR-reactions were performed either by the QuikChange site-specific mutagenesis kit from Stratagene, the Expand high fidelity PCR system kit from Roche or the Phusion® High-Fidelity DNA Polymerase kit from New England Biolabs, according to the manufacturer’s recommendations.

### Plasmid constructions and vector descriptions

The plasmid pTA13 [10] was used for construction of pFS7. This plasmid harbours the *Pm* promoter with *bla* as reporter gene and the gene coding for *xylS* behind the natural *Ps2* promoter in combination with a minimal RK2 replicon. A new NdeI-site was introduced downstream of *xylS* by site-specific mutagenesis. The *luc*-gene was amplified from pKT1 [29] with NdeI- and AgeI- flanking ends and inserted downstream of *xylS*. The NdeI-site was removed in a subsequent step by cloning of a PCR-amplified NcoI-*xylS*-BbsI-fragment from pTA13 into the new vector. A spacer was inserted upstream of the overlapping stop-start codon by site-specific mutagenesis to eliminate the function of a potential Shine-Dalgarno site at the 3′-end of *xylS*. Different variants of *xylS* were inserted via site-specific mutagenesis or insertion of annealed oligonucleotides upon digestion with suitable enzymes.

For construction of pFZ2A, *xylS* and its *Ps2* promoter were PCR-amplified with AgeI- and EcoRI-flanking sites from pTA13 [10] and inserted into pBBR1-MCS-5 [33].

To obtain pFZ2B1 the *Pb* promoter part of pMS119 delta *chnE*[34] was PCR-amplified with BstZ171- and NdeI- flanking ends and cloned into pTA16 [28]. The *chnR* part of pMS119 delta *chnE* was PCR-amplified with AgeI- and SacI-flanking ends and integrated into the plasmid which already contained the *Pb* promoter. The resulting plasmid was named pRL17A. *xylS* was cloned behind the *Pb* promoter in this plasmid by digestion with KpnI and NcoI. An XhoI-BamHI-fragment was then cloned into vector pBBR1-MCS-5 [33], resulting in plasmid pFZ2B1.

In pFZ2B2 and pFZ2B3 the promoter in front of the gene *chnR*, coding for the regulator protein of *Pb* in pFZ2B1, was exchanged by two of the constitutive promoters (Anderson-collection, BBa_J23105 = A, BBa_J23103 = B) from the Registry of Standard Biological Parts [35]. For this one-step sequence- and ligation-independent cloning [38] was used. The two promoters increase levels of ChnR and thus result in stimulated expression from *Pb* (unpublished results).

pET16b.xylS is a plasmid based on pET16b (Novagen), where the ampicillin resistance gene was exchanged by a tetracycline resistance gene and *xylS* was inserted as NdeI-BamHI fragment behind the T7 promoter.

pFS15 is a derivative of pTA13, where *xylS* has been removed by digestion with AgeI and SacI and insertion of a short linker.

### Test of XylS expression via host ampicillin tolerance

To monitor changes in XylS expression indirectly, *bla* under control of the *Pm* promoter was used as a reporter gene. Higher expression from *Pm* leads to increased β-lactamase production and corresponding host ampicillin tolerance in a nearly linear relationship with the ampicillin concentrations used in this study [32]. Changes in XylS expression will consequently lead to varying levels of expression from *Pm* in the presence of *m*-toluate*,* which can easily be characterized by simply plating cells on agar medium supplied with a gradient of increasing levels of ampicillin. Thus the levels of *bla*-expression will indirectly reflect the level of XylS being expressed. For ampicillin tolerance testing cultures were grown in LB medium in 96-well plates (at least three replicates per sample) overnight, diluted in fresh LB (1:10^4^), plated on agar medium with a pin replicator, and incubated at 30°C for 48 hours. The plates were then inspected visually. The highest ampicillin concentration on which growth occurred for the majority of the replicates was treated as maximum ampicillin tolerance, while the lowest concentration in test at which no growth was observable is indicated as error bar in the corresponding figures. All ampicillin tolerance testing experiments were performed at least twice. Ampicillin concentrations varied from 5 μg mL^-1^ to 4500 μg mL^-1^.

### Test of XylS expression levels using a synthetic operon and luciferase assay

XylS amounts could be measured more directly via luciferase activity in all constructs based on pFS7. Luciferase activity was measured using the Luciferase Assay System from Promega, according to the manufacturer’s protocol. The luminometer used was a GloMax 20/20 (Promega). Strains were grown as described above.

### RNA isolation, cDNA synthesis and qRT-PCR

Transcript amounts were determined by two-step quantitative real-time reverse-transcriptase polymerase chain reaction (qRT-PCR). RNAqueous (Ambion) was used for total RNA isolation. Isolated RNA was treated with Turbo DNAse (Ambion) and reverse transcription was performed using a first-strand cDNA synthesis kit with random pd(N)6 primers (Amersham Biosciences). PCR was carried out in the presence of Power SYBR Green PCR Master Mix (Applied Biosystems) using a 7500 Real Time PCR system (Applied Biosystems). During PCR samples were heated to 95°C for 10 min, followed by 40 cycles of amplification (95°C for 15 s; 60°C for 1 min). Results were analysed by 7500 system software v1.3 using the 2^-∆∆CT^ method [39]. Primers were designed using Primer Express software (Applied Biosystems). For *xylS* primers 5′-TGTTATCATCTGCAAATAATACTCAAAGG-3′ and 5′-GCCCGGCGCAAAATAGT-3′ were used. 16S rRNA was used as endogenous control with the primer pair 5′-ATTGACGTTACCCGCAGAAGAA-3′ and 5′-GCTTGCACCCTCCGTATTACC-3′.

### Protein analysis by SDS-PAGE

For SDS-PAGE analysis cells were grown in a volume of 25 mL. Cultures containing plasmid pET16b.xylS were induced with 0.5 mM IPTG or grown in the absence of inducer. After centrifugation the pellets were washed in 0.9% NaCl. 100 mg pellet (wet weight) were resuspended in 0.5 mL lysis buffer (50 mM Tris–HCl, pH 8.0, 1 mM EDTA, pH 8.0, 20% sucrose), 1 mg lysozyme and 62.5 U mL^-1^ benzonase nuclease (Sigma) were added and samples were left with shaking at room temperature for 2 hours. After centrifugation (13.000 rpm, 8 min) the supernatant was used as soluble fraction, while the pellet was resuspended in 0.5 mL SDS-PAGE running buffer, giving the insoluble fraction. Protein gels were run under denaturing conditions using ClearPAGE 10% gels and ClearPAGE SDS-R Run buffer (C.B.S. Scientific) followed by staining with Coomassie Brilliant blue R-250 (Merck).

## Competing interests

The authors declare that they have no competing interests.

## Authors’ contributions

All authors were involved in the experimental design and FZ and RL stood for the practical execution. All authors contributed to the writing of the manuscript. All authors read and approved the final manuscript.
